# Python Executable Script for Estimating Two Effective Parameters to Individualize Brain-Computer Interfaces: Individual Alpha Frequency and Neurophysiological Predictor

**DOI:** 10.3389/fninf.2016.00022

**Published:** 2016-06-22

**Authors:** Luz María Alonso-Valerdi

**Affiliations:** Escuela de Ingeniería y Ciencias, Tecnológico de MonterreyMexico City, Mexico

**Keywords:** brain-computer interface (BCI), motor imagery, individual alpha frequency, neurophysiological predictor, BCI illiteracy, electroencephalographic signals

## Abstract

A brain-computer interface (BCI) aims to establish communication between the human brain and a computing system so as to enable the interaction between an individual and his environment without using the brain output pathways. Individuals control a BCI system by modulating their brain signals through mental tasks (e.g., motor imagery or mental calculation) or sensory stimulation (e.g., auditory, visual, or tactile). As users modulate their brain signals at different frequencies and at different levels, the appropriate characterization of those signals is necessary. The modulation of brain signals through mental tasks is furthermore a skill that requires training. Unfortunately, not all the users acquire such skill. A practical solution to this problem is to assess the user probability of controlling a BCI system. Another possible solution is to set the bandwidth of the brain oscillations, which is highly sensitive to the users' age, sex and anatomy. With this in mind, NeuroIndex, a Python executable script, estimates a neurophysiological prediction index and the individual alpha frequency (IAF) of the user in question. These two parameters are useful to characterize the user EEG signals, and decide how to go through the complex process of adapting the human brain and the computing system on the basis of previously proposed methods. NeuroIndeX is not only the implementation of those methods, but it also complements the methods each other and provides an alternative way to obtain the prediction parameter. However, an important limitation of this application is its dependency on the IAF value, and some results should be interpreted with caution. The script along with some electroencephalographic datasets are available on a GitHub repository in order to corroborate the functionality and usability of this application.

## Introduction

A brain-computer interface (BCI) is a system that attempts to establish communication between the human brain and a computing system, in order to achieve interaction between an individual and his environment without using the brain output pathways (nerves and muscles). At the initial stages of BCI development, the systems were designed for people suffering from severe neuromuscular deficits provoked by disorders such as multiple sclerosis, or spinal cord injuries. Recently, the interest in BCI research has grown exponentially and current applications include entertainment, rehabilitation, diagnosis, treatment, and intelligent housing systems (Hassanien and Azar, 2014).

**Figure 1:**
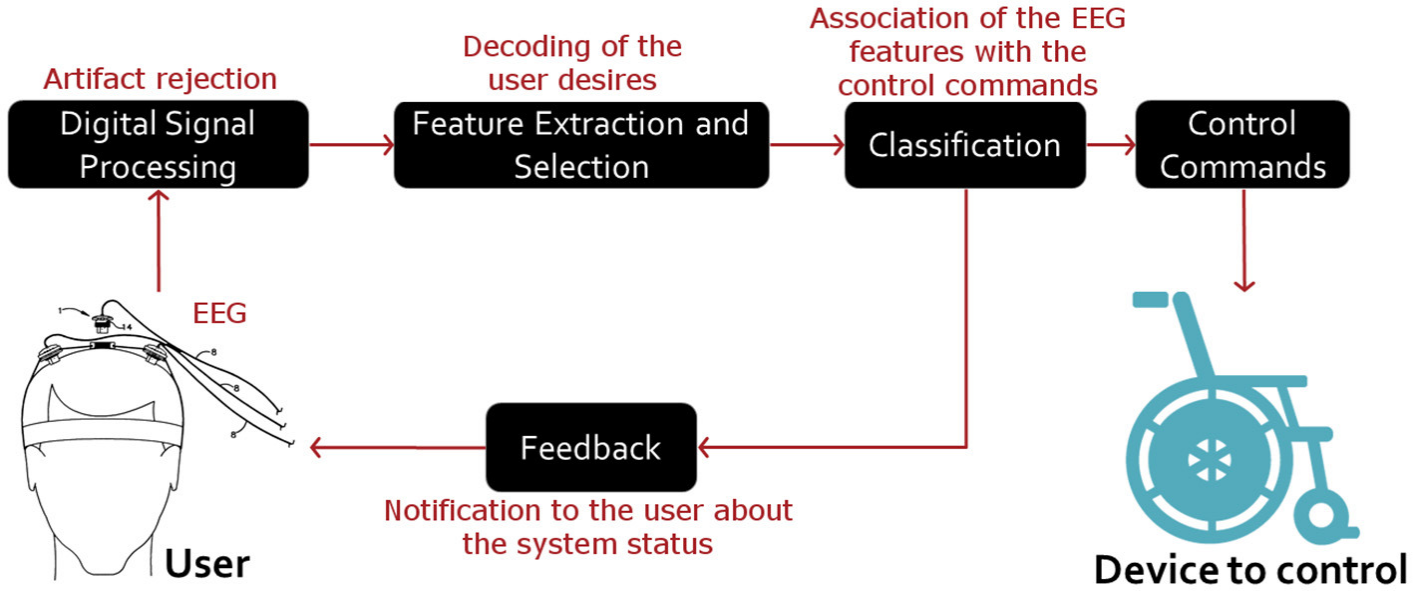
**General structure of EEG-based BCI systems**. In these systems, internal (such as other electrophysiological activity) and external (such as the line source) artifacts are first rejected. Then, the user desires are decoded by extracting signal features that can reflect the control tasks in use. Finally, such features are related to the control commands of the device of interest. Note that the user is notified about the system status at any time.

A BCI system essentially functions as follows. Firstly, the brain signals are sensed, amplified and processed. Such signals are typically recorded using electroencephalography (EEG), a non-invasive method that measures the electrical activity of the cerebral cortex. Secondly, the system seeks and extracts useful electrophysiological features of the EEG signals, which reflect the user desires of controlling the system. Finally, the system associates the meaningful EEG features with specific control commands of a target device (Lotte and Jeunet, 2015). Note that the user is notified about the system status at any time. See Figure 1.

The electrophysiological neuro-mechanisms employed to establish brain-computer communication generally fall into three broad categories: endogenous, exogenous, and combination thereof. The endogenous mechanism depends on the quantification of brain oscillations that are modulated through specific mental tasks, including motor imagery (MI), mental calculation, or association of imaginary words. The exogenous mechanism is based on the detection of evoked potentials, resulting from auditory, visual, or tactile stimuli (Bashashati et al., 2007; Fatourechi et al., 2007). Both mechanisms are referred to as *control task*. One commonly used control task is MI, which refers to the kinaesthetic imagination of a part of the body that is usually a hand, a foot or the tongue. Imaginary movements synchronize and desynchronize the neural activity of the sensory-motor cortex, almost at the same extend as actual movements. The neural de-synchronization events that characterize MI activity are alpha (≈8–12 Hz) and beta (≈16–24 Hz) band desynchronization, locally restricted to the contralateral sensory-motor cortex (Neuper et al., 2006; Szurhaj and Derambure, 2006; Farina et al., 2013).

The modulation of the EEG signals through MI related control tasks is a skill that must be acquired, what means that users need to be trained. Users modulate their EEG signals at different frequencies and different levels due to training duration, aptitude, age, sex, and anatomy. Some users are unfortunately unable to modulate their EEG signals, even after training (Blankertz et al., 2010). The characterization of the user brain signals, and the anticipation of the user aptitude to control a MI based BCI, can be two feasible solutions to achieve brain-computer communication by means of MI related control tasks.

So far, some neurophysiological methods have been proposed to adjust the bandwidth of the brain oscillations according to the nature of the user EEG signals, and some others have been suggested to predict the user performance (Grosse-Wentrup and Scholkopf, 2012; Hammer et al., 2012; Zhang et al., 2015). As far as I know, no implementations of those methods are available yet. On this basis, a Python executable script called NeuroIndex was programmed with two main objectives:

(1) To find the particular frequency oscillation of the sensory-motor rhythms of every user according to the individual alpha frequency (IAF) parameter, and thus preventing anatomic, age, and gender conflicts.
(2) To estimate a neurophysiological indicator based on the previously adjusted sensory-motor rhythms, which could predict the user aptitude for modulating the EEG signals by using MI related control tasks.

As was aforementioned, NeuroIndex is an executable script written in Python programming language. The script was built on top of *Numpy* and *Scipy* (Van Der Walt et al., 2011), in addition to *Matplotlib* (Hunter, 2007), a plotting library, and *lmfit* (Newville and Stensitzki, 2015), a curve fitting library. By double-clicking the script, a command prompt window will be opened and three items of information must be provided: (1) sample rate at which EEG signals were recorded, (2) a text file (or mat-file) containing an EEG recording in eyes-open (EO) condition for 3 min, and (3) a text file containing an EEG recoding in eyes-close (EC) condition for 3 min. Note that the default EEG layout corresponds to the 10/10 system with 64 channels enumerated according to the BIOSEMI Company^1^. Once the program is run, two figures will be created in the same folder in which text files are localized: one corresponding to the IAF, and the other corresponding to the neurophysiological predictor.

## Software description

As was introduced in the previous section, one of the major drawbacks of BCI technology is the considerable variation intra- and inter-subject. Therefore, several methods have been proposed in order to personalize a BCI system, and thus minimizing subject variance effects. NeuroIndeX is an application that determines the IAF and a neurophysiological indicator. The IAF is a parameter utilized to tune the frequency oscillation of the sensory-motor rhythms, and the neurophysiological indicator is a prediction index that quantifies the user aptitude for controlling a MI based BCI. The procedure adopted for determining these two parameters is fully described below.

**Figure 2:**
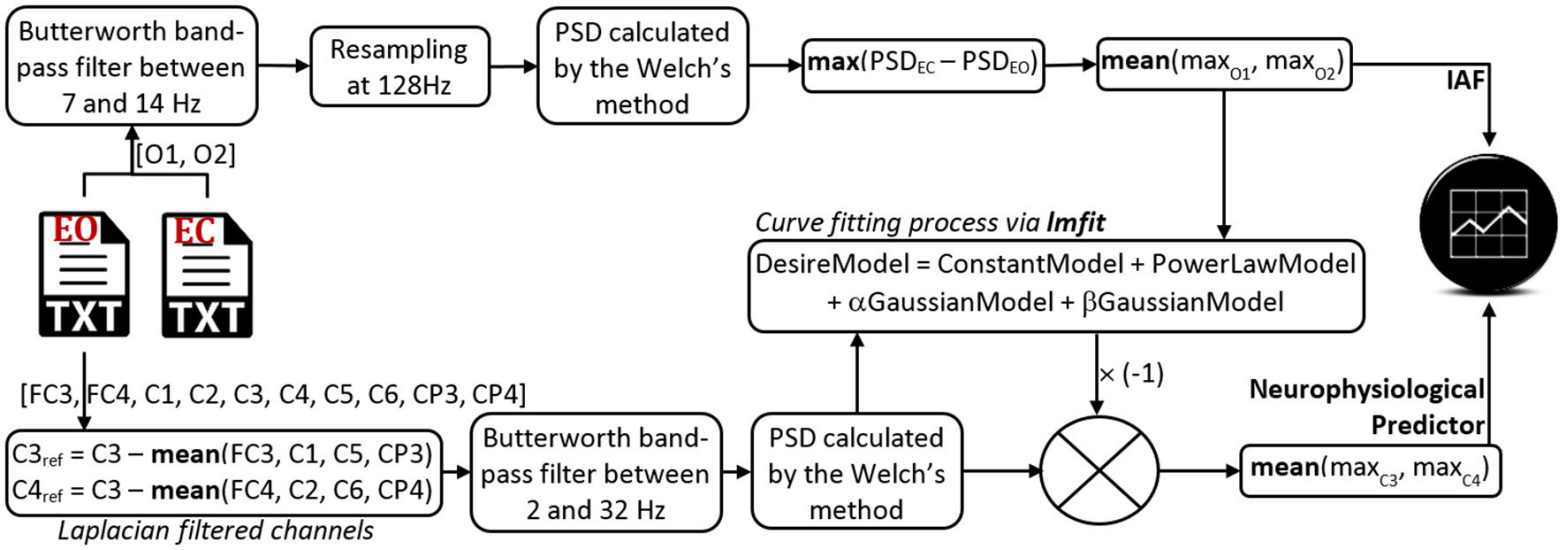
**Architecture of the NeuroIndeX software**. This application is a Python executable script that estimates the IAF and the neurophysiological prediction index. Only two 3-min-long EEG recordings are necessary, one in EO condition and another one in EC condition. The software and some datasets for testing purposes are available in a GitHub repository.

To obtain the IAF, NeuroIndex follows the method proposed by Posthuma et al. (2001) and two 3-min-long EEG recordings in EC and EO conditions are necessary. The IAF is obtained from two occipital channels (O1 and O2), wherefrom the power spectral density (PSD) is estimated by applying the Welch's method, and using 4-s-long epochs (Figure 2). Once NeuroIndex has determined the IAF, this value can be used to define two narrow alpha bandwidths in the following way: from {IAF−2} to {IAF}, and from {IAF} to {IAF+2}. Klimesch (1999) recommended the individualization of the alpha band frequency around IAF and within narrow bands of around 2 Hz, since alpha band changes in accordance with the mental state, age and gender of the individual in question. As a result, the frequency oscillation of the alpha rhythms is set to the user signal features, instead of making use of the usually preset frequency bands.

To estimate the neurophysiological predictor, NeuroIndex makes use of the method proposed by Blankertz et al. (2010). The method requires a 2-min-long EEG recording in EO condition. The general procedure consists in calculating the PSD of two central recording sites: C3 and C4. Thereafter, both PSDs are fitted through a mathematical model based on a constant, a power law function and two probability density functions: one concerning the alpha band peak, and another one concerning the beta band peak (Equation 1). Once an optimal model has been obtained, the prediction index is calculated by the maximum difference between that model and the power law function (Figure 2). As larger the index is, the user aptitude is higher. The early identification of low aptitude users avoids long and tiring training sessions spent in a pointless user-system adaptation.

(1) $$\begin{array}{l} Model_{PSD}=f_{1}+f_{2}\left(x;A_{2},k_{2}\right)+f_{3}\left(x;A_{3},\mu ,\sigma \right)\\ \;=k_{1}+A_{2}x^{k_{2}}+\frac{A_{3}}{\sigma \sqrt{2\pi }}e^{-\left(x-\mu^{2}\right)/2\sigma^{2}\;} \end{array}$$

## Illustrative examples

In order to exemplify the functionality of NeuroIndex, I have made use of a database created by Alonso-Valerdi and Sepulveda (2014). The database contains EEG and Electrocardiography (ECG) recordings of 11 participants who were exposed to nine different scenarios. In every scenario, the sensory-cognitive workload was gradually increased so as to achieve a better user-system adaptation. From the nine scenarios, seven of them were used to adapt the user with the platform in use. The rest of them were employed to test the user aptitude for establishing brain-computer communication under simulated living situations. At the beginning of each experimental session, all the participants were asked to stay in two conditions: EC and EO. Both conditions were recorded for 3 min at 128 Hz, while the nine scenarios were recorded at 256 Hz. In any case, EEG and ECG signals were recorded using an ActiveTwo amplifier and ActiView software (BIOSEMI Company—The Netherlands). To register the EEG signals, sixty one recording sites in line with the 10/10 System were employed, while the lead I of the Einthoven triangle was used to register the ECG signal. The experimental procedure undertaken to obtain the recordings was approved by the Ethics Committee of the University of Essex, and written informed consent from all the participants was obtained as well. From the entire database, only EC and EO conditions were taken to illustrate the usability of the present program. Note that EEG signals are required for the NeuroIndex software, whereas the ECG signal is only necessary for the evaluation of the software, which will be discussed in Section System Performance.

According to the performance of each participant reported by Alonso-Valerdi and Sepulveda (2014), two of the 11 participants (P1–P2) were unable to establish brain-computer communication. Five of the 11 ones (P3–P7) were able to establish communication in one of the two simulated living situations. The rest of them (P8–P11) established communication in both simulated living situations. The EEG recordings of P2, P6, P7, and P11 (one participant from two categories and two participants from one of the categories) were selected to show the NeuroIndeX functionality. The EEG recordings of these four participants, as well as the NeuroIndeX software output of the eleven participants, are available on a GitHub repository^2^.

**Figure 3:**
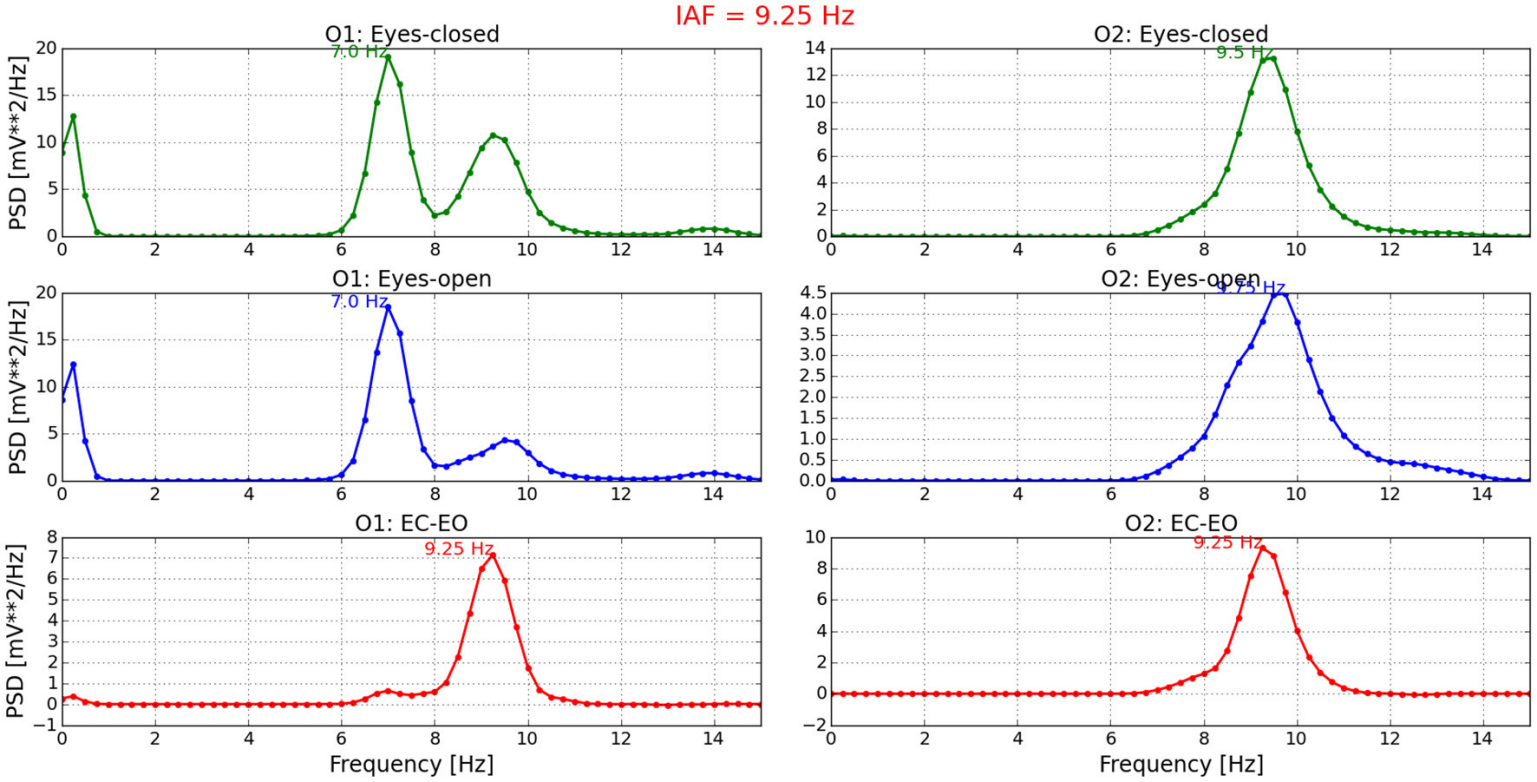
**Individual alpha frequency estimated for P11**. On the left side, the PSD of channel O1 is presented in three conditions: EC, EO, and EC-EO. Similarly, the PSD of channel O2 is shown on the right side. The average of the third condition (EC-EO) in both channels (red line) is the IAF and the NeuroIndex output. This values titles the figure.

Figure 3 presents the IAF of P11. As can be seen from the figure, the whole process is graphically set out. At the top, PSD obtained from O1 and O2 channels in EC condition is shown. In the middle, the PSD in EO condition is provided. At the bottom, the difference of both conditions is illustrated and the average of the corresponding peak values is finally the IAF. For the rest of the participants, similar graphs were obtained. Those outcomes can be revised on the repository.

**Figure 4:**
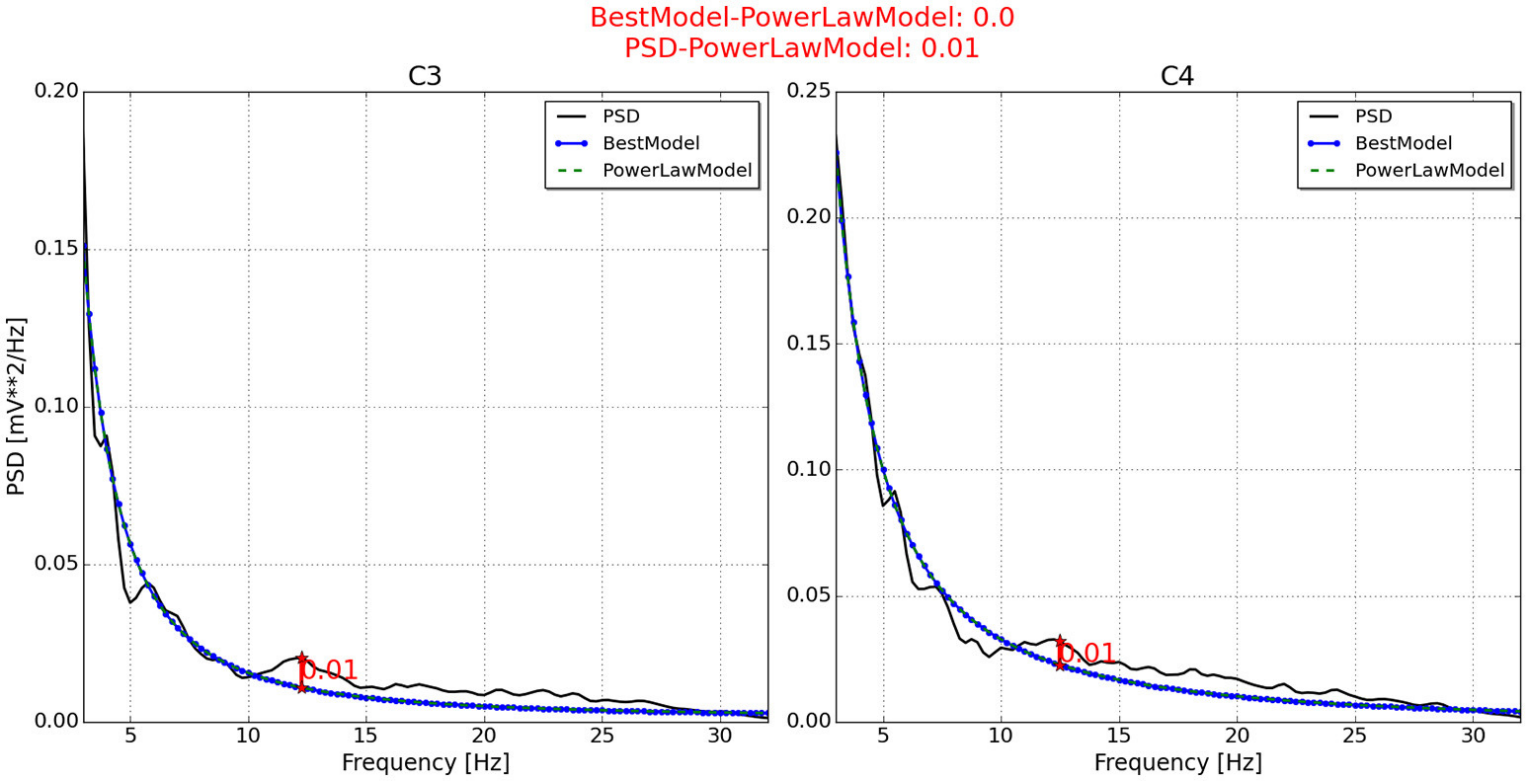
**Neurophysiological predictor determined for P2**. The best model (blue line) of the PSD (black line) is very similar to the power law function (green and dotted line). Therefore, the neurophysiological index is calculated by differentiating the PSD and the power law function, instead of using the best model as Blankertz et al. (2010) proposed.

**Figure 5:**
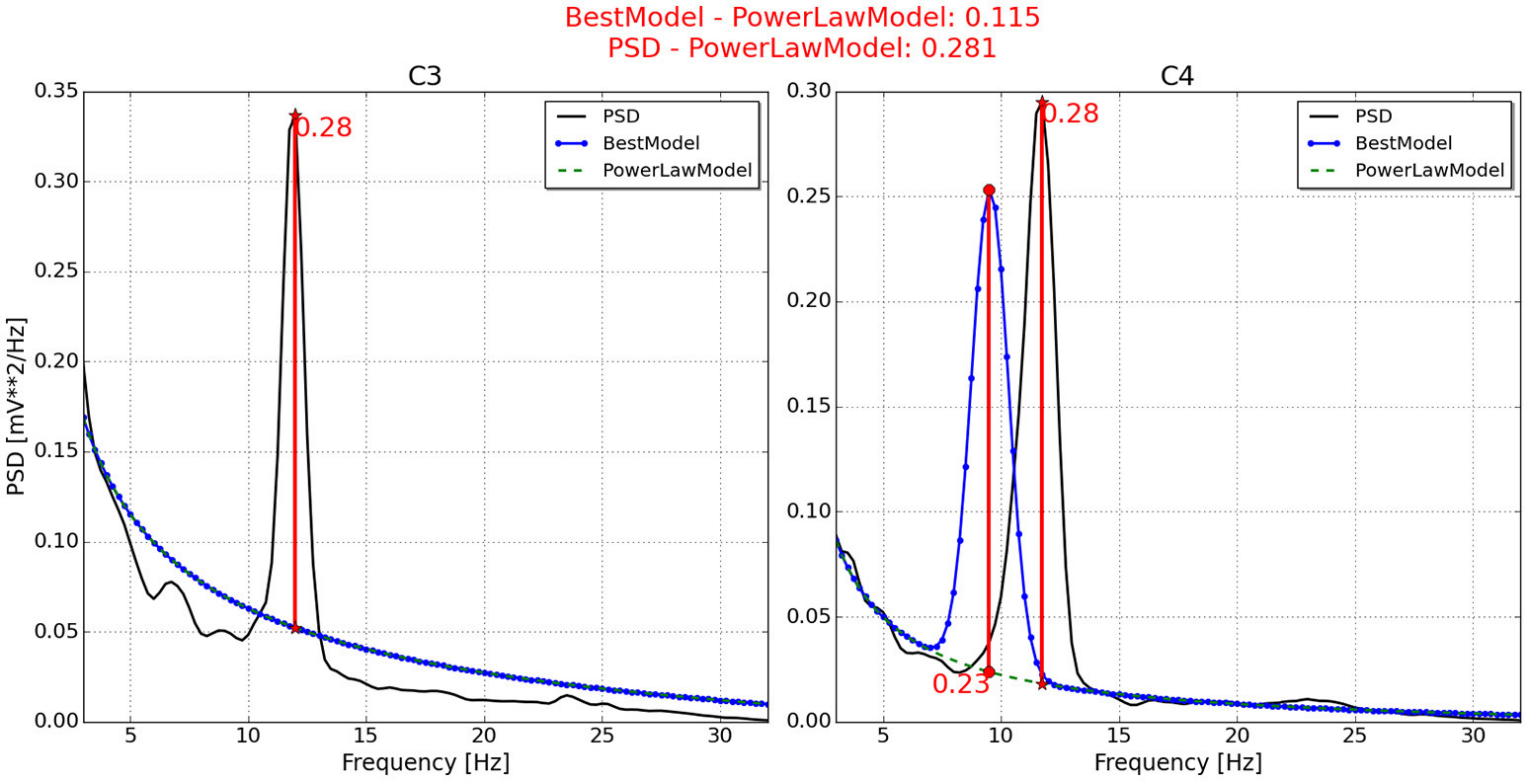
**Neurophysiological predictor determined for P6**. The best model (blue line) of the PSD (black line) is very similar to the power law function (green and dotted line) on channel C3, while it is shifted to the left on channel C4. As a consequence, the feasible neurophysiological index is that calculated using the PSD, instead of the best model.

**Figure 6:**
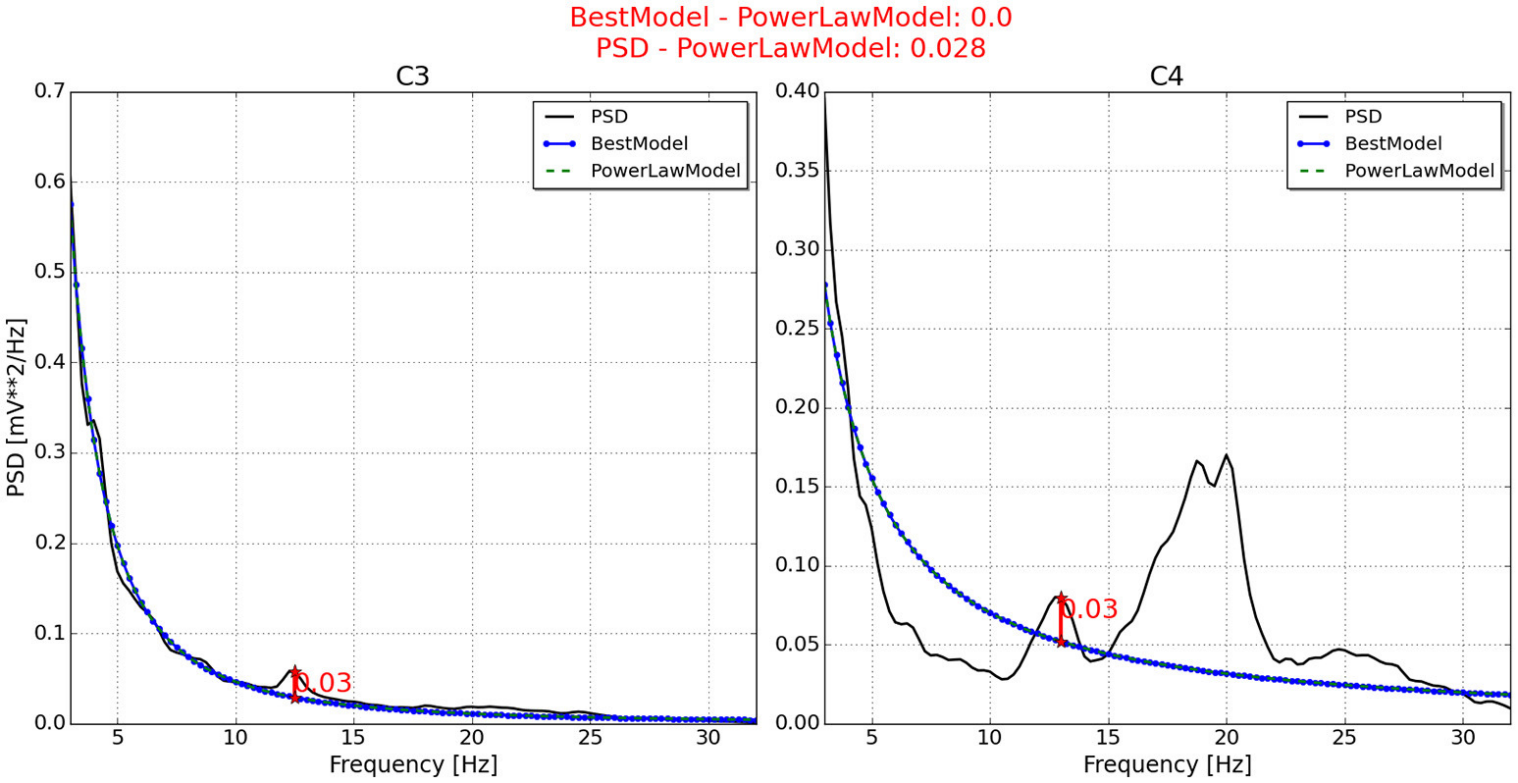
**Neurophysiological predictor determined for P7**. The best model (blue line) of the PSD (black line) is very similar to the power law function (green and dotted line). In addition, the PSD waveform is very unusual, resulting in an incorrect neurophysiological index.

**Figure 7:**
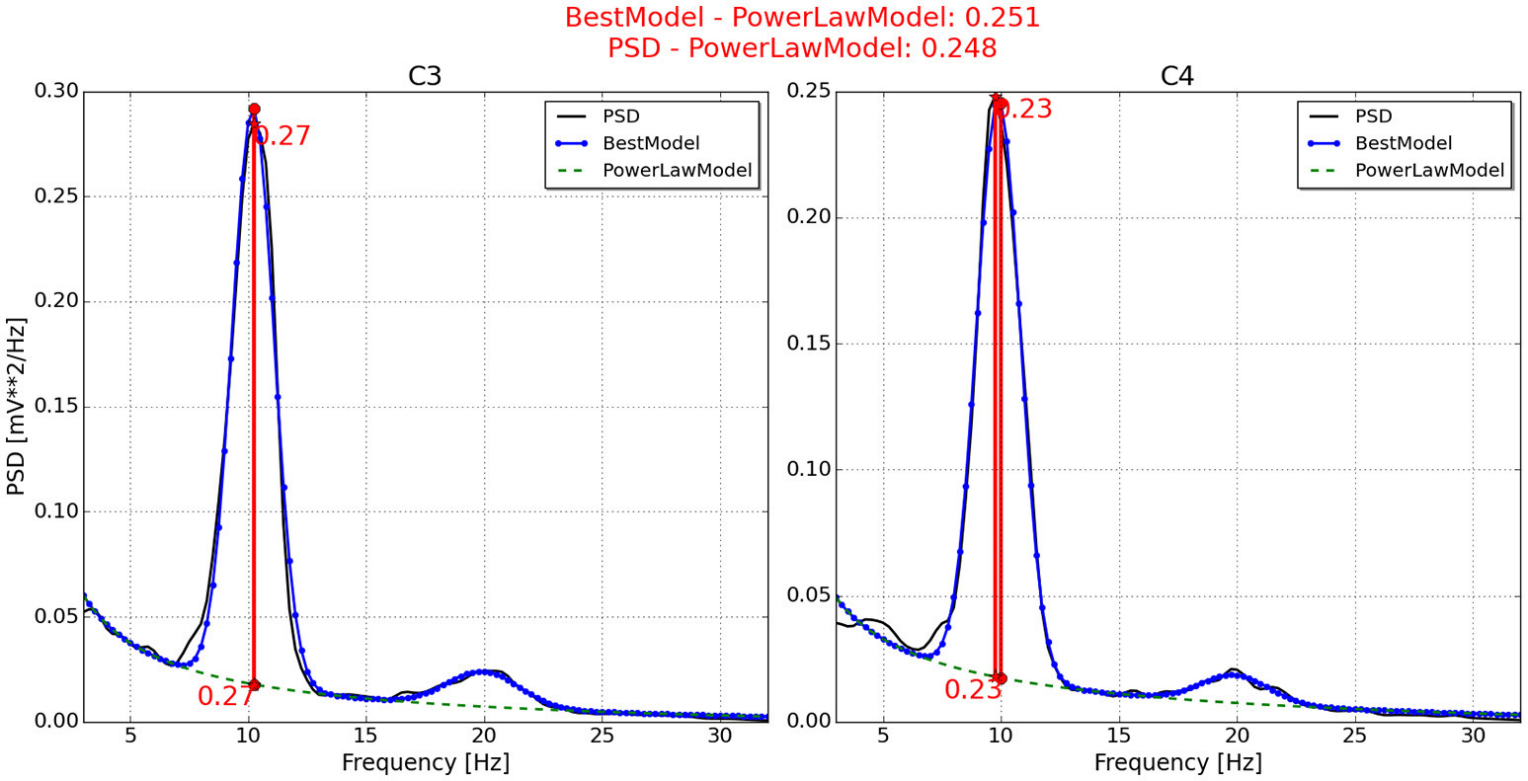
**Neurophysiological predictor determined for P11**. The best model (blue line) of the PSD (black line) is nicely fitted, producing very similar neurophysiological indexes. One index is estimated by the differentiation between the best model and the power law function (green dotted line). Another index is estimated by the differentiation between the PSD and the power law function.

With respect to the neurophysiological predictor, the values for P2, P6, P7, and P11 are respectively presented in Figures 4–**7**. As can be seen from Figure 4, the best model and the power law model for the PSD of P2 are overlapped. As a result, the neurophysiological predictor cannot be estimated as was proposed by Blankertz et al. (2010), i.e., max (BestModel—PowerLawModel). Alternatively, NeuroIndex provides the maximum difference between the original PSD and the power law model. The neurophysiological predictor has a very small value, which agrees with the poor performance of P2 reported by Alonso-Valerdi and Sepulveda (2014). For P6 (Figure 5), NeuroIndex failed to determine an appropriate model that could be fitted to the alpha band peak on C3, giving a smaller neurophysiological indicator in comparison to the alternative value provided by the software. Note that the alternative value agrees with the overall performance of P6. For P7 (Figure 6), NeuroIndex was completely unable to model the PSD, resulting in an incorrect calculation of the neurophysiological predictor. In this case, the user performance could not be predicted. Lastly, in Figure 7, the software output for P11 is provided. The figure illustrates a very nice curve fitting in which both values are quiet similar. This result perfectly agrees with the performance of P11, who was a user able to establish efficient brain-computer communication.

## System performance

To evaluate the NeuroIndex performance, other similar applications were searched. However, only an EEGLAB plugin to analyze the IAF was found (Goljahani et al., 2014), while no software was found to estimate the neurophysiological predictor proposed by Blankertz et al. (2010). The application developed by Goljahani et al. (2014) is an EEGLAB plugin to analyze individual EEG alpha rhythms using the channel reactivity-based method proposed by Goljahani et al. (2012). This method measures an overall IAF by identifying the EEG power variations across different channels, while the subject in question is performing specific tasks. Herein, the computation of the IAF depends on the dynamic neurophysiological process of the brain, where reference and test mental states are compared. As this EEGLAB plugin requires event-related data and its purpose is not consistent with that of NeuroIndex, it was not possible to obtain IAF values from the datasets at hand in order to compare them with the NeuroIndex output.

In the light of the above issues, it was decided to determine theoretical IAFs and compare those values with the NeuroIndex output. As the neurophysiological predictor index depends on the IAF value, the evaluation of the application could be considered objective.

### Theoretical IAF values

According to Klimesch (2013), there exists a brain-body interaction that may be described as a complex system that couples and decouples on the basis of a specific harmonic frequency structure mathematically defined by

(2) $$f_{d}(i)=s^{*}2^{i}\text{ where }i=0,1,2,3,...$$

Klimesch suggested that ***s*** is the cardiac frequency in Hz and can be found when *i* = 0. Having the cardiac frequency, the central frequency of the typical EEG frequency bands (δ, θ, α, β, γ) can be obtained by evaluating Equation (2). For example, if *s* = 1.25 Hz (75 beats per minute), the EEG frequency bands are defined as follows

$$\begin{array}{l} f_{d}(1)=1.25^{*}2^{1}=2.5Hz=\delta \\ f_{d}(2)=1.25^{*}2^{2}=5.0Hz=\theta \\ f_{d}(3)=1.25^{*}2^{3}=10Hz=\alpha \\ f_{d}(4)=1.25^{*}2^{4}=20Hz=\beta \\ f_{d}(5)=1.25^{*}2^{5}=40Hz=\gamma \end{array}$$

On this basis, the cardiac frequency was calculated using the lead I of the Einthoven triangle, and thus estimating the theoretical IAF by Equation (2). To calculate the cardiac frequency, the ECG signal was first high-pass filtered at 0.1 Hz; QRS complexes were then detected by the *Pan-Tompkins* method (Pan and Tompkins, 1985) implemented by Sedghamiz (2014); and the NN intervals (time distance between two adjacent QRS complexes) were finally determined. Those NN intervals were averaged and inversed, thereby obtaining the cardiac frequency (***s***). To estimate the theoretical IAF, Equation (2) was evaluated when *i* = 3.

### Comparison between theoretical and empirical IAFs

**Figure 8:**
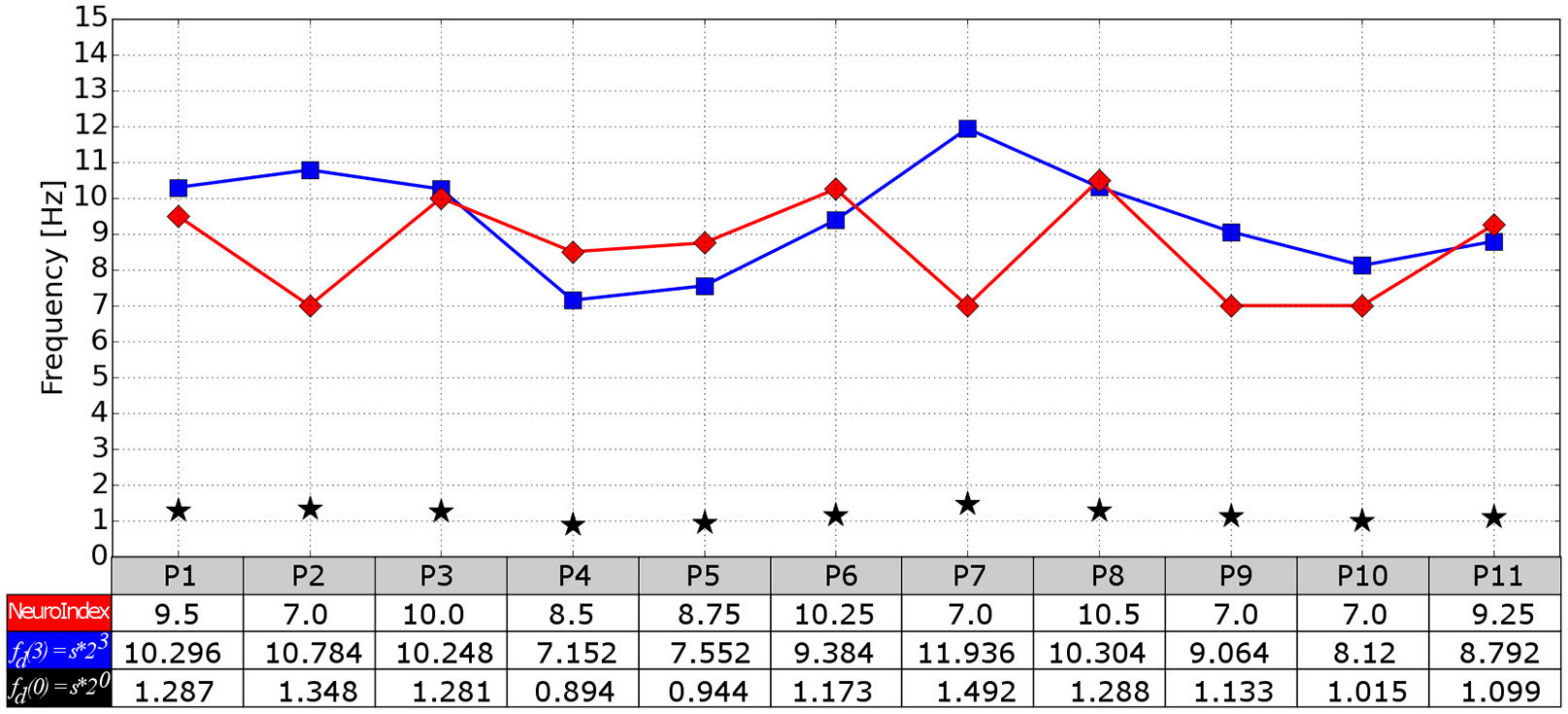
**Comparison between the NeuroIndex output (in terms of IAF) and IAF values theoretically determined**. Three values per participant are provided: The NeuroIndex output (red line), the IAF values calculated in terms of the cardiac frequency (blue line), and the cardiac frequency (black star markers).

The cardiac frequency, the theoretical IAF and the NeuroIndex output are compared in Figure 8. By applying the Student's *t*-test, the theoretical IAFs and the NeuroIndex outputs are not significantly different (*p* = 0.2204). However, a difference of 1 Hz in the process of determining the EEG frequency bands must be considered carefully. As can be seen from the figure, P2 and P7 show the largest different between theoretical and empirical IAFs. In both cases, the alpha peaks of the PSD (P2: Figure 4, P7: Figure 6) are between 10 and 15 Hz. It seems that the theoretical IAFs (P2: 10,784 Hz, P7: 11,936 Hz) may be a better approach in comparison to the NeuroIndex outputs (P2: 7 Hz, P7: 7 Hz), which were obtained from EC-EO conditions and using the method proposed by Posthuma et al. (2001). By replacing the empirical IAFs for the theoretical one, the PSD fitting can be improved, and in turn, the neurophysiological index could be accurately detected. It seems possible that brain-body coupling model proposed by Klimesch (2013) may improve the NeuroIndex performance.

## Discussion

One of the main shortcomings of BCI technology has been the substantial variation of the user performance. A way to deal with such variation is the localization of the individual frequency oscillation of the sensory-motor rhythms using the IAF value (specifically alpha band rhythms), and the identification of possible illiterate users in accordance with the neurophysiological predictor. The measurement of these two parameters before the user training may help to adjust the system in line with the user signal features, and can also allow to select another type of BCI paradigms whenever necessary.

Most of the BCI community effort has been put in designing applications for online and offline signal processing. Up to now, a wide variety of proficient BCI packages are available, including BCI2000, BCILab, OpenViBe, TOBI, and BioSig. However, far too little attention has been paid to the software development concerning the user evaluation before the human-machine adaptation. In this regard, NeuroIndeX is an easy application that can be used to characterize the user EEG signals, and decide how to go through the complex process of adapting the human brain and the computing system. Furthermore, NeuroIndeX is not only the implementation of previously proposed methods, but it also complements two different methods and provides an alternative way to obtain the prediction parameter. On the one hand, the calculation of the neurophysiological predictor is based on the IAF. On the other hand, if a mathematical model cannot be obtained by means of a power law model and a probability density function, NeuroIndeX anyway proposes an indicator by employing the power law model and the original PSD. Nonetheless, an important limitation of this application is its dependency on the IAF value. For instance, the neurophysiological index of P7 (Figure 6) was wrongly estimated owing to an inappropriate PSD fitting. As the peak values of the PSD are far from the IAF value determined for the participant (7 Hz, this can be revised in the repository), the brain-body coupling model proposed by Klimesch (2013) may be a better option to determine an accurate IAF value, and in turn, an appropriate PSD fitting. Therefore, the software outputs need to be interpreted with caution.

Lastly, it is worth noting that the determination of these two neurophysiological indexes is not limited to the BCI community interest. The analysis of EEG signals is nowadays a key issue in many other research fields such as Psychology, Medicine, Computer Sciences, and obviously Neurosciences. As a case in point, the study of the level of synchronization of the neural networks is very important to understand the sensory-cognitive processing of the human brain. This study can be much more fruitful if the frequency oscillation of the brain rhythms of interest is well-localized.

## Conclusions

NeuroIndeX is an application completely written in Python, which pursuits to improve the applicability of MI based BCIs by characterizing the user EEG signals before undertaking the human-machine adaptation process. Such characterization is based on estimating the IAF according to Posthuma et al. (2001), and the neurophysiological predictor proposed by Blankertz et al. (2010). With these two parameters, the appropriate adjustment of the sensory-motor rhythms and the selection of an adequate BCI paradigm are possible. The present application only requires two 3-min- long EEG recordings in two conditions: EO and EC. The sample rate of the recordings must be provided as well. The Python script, EEG testing data, a brief instruction manual and some examples of the software output are available on https://github.com/LuzAlondra/BrainComputerInterfaces. Regardless of the wide variety of BCI software available at the moment, it seems that no current applications for characterizing the user EEG signals are offered yet. Therefore, NeuroIndex might be a very helpful tool to optimize the complex process of adapting the human brain and the computing system.

## Author contributions

LA developed the presented software, evaluated the functionality of the software and wrote the manuscript.

### Conflict of interest statement

The author declares that the research was conducted in the absence of any commercial or financial relationships that could be construed as a potential conflict of interest.

## Footnotes

^1^http://www.biosemi.com/headcap.htm.

^2^https://github.com/LuzAlondra/BrainComputerInterfaces.
